# Batch Transfer Printing of Small-Size Silicon Nano-Films with Flat Stamp

**DOI:** 10.3390/mi12101255

**Published:** 2021-10-16

**Authors:** Wenping Cao, Guochang Liu, Jinwei Miao, Guojun Zhang, Jiangong Cui, Yuhua Yang, Changde He, Wendong Zhang, Renxin Wang

**Affiliations:** State Key Laboratory of Dynamic Testing Technology, North University of China, Taiyuan 030051, China; caowenping517@163.com (W.C.); liuguochang@live.com (G.L.); miaojinwei96@foxmail.com (J.M.); zhangguojun1977@nuc.edu.cn (G.Z.); cuijiangong99999@163.com (J.C.); yangyuhua407@163.com (Y.Y.); hechangde@nuc.edu.cn (C.H.); wdzhang@nuc.edu.cn (W.Z.)

**Keywords:** transfer printing, PDMS, PI, adhesion, bonding

## Abstract

Silicon nano-film is essential for the rapidly developing fields of nanoscience and flexible electronics, due to its compatibility with the CMOS process. Viscoelastic PDMS material can adhere to Si, SiO_2_, and other materials via intermolecular force and play a key role in flexible electronic devices. Researchers have studied many methods of transfer printing silicon nano-films based on PDMS stamps with pyramid microstructures. However, only large-scale transfer printing processes of silicon nano-films with line widths above 20 μm have been reported, mainly because the distribution of pyramid microstructures proposes a request on the size of silicon nano-films. In this paper, The PDMS base to the curing agent ratio affects the adhesion to silicon and enables the transfer, without the need for secondary alignment photolithography, and a flat stamp has been used during the transfer printing, with no requirement for the attaching pressure and detaching speed. Transfer printing of 20 μm wide structures has been realized, while the success rate is 99.3%. The progress is promising in the development of miniature flexible sensors, especially flexible hydrophone.

## 1. Introduction

Flexible electronics are formed by organic or inorganic electronic devices, combined with the flexible substrate in a certain way. In recent years, flexible electronics has been widely used in areas such as biology [1,2,3,4], information [5,6], and energy [7,8]. Compared to conventional electronics, flexible electronics can be used more widely, operate in almost all types of environments, and adapt to the deformation of the equipment to some extent [9].

In the field of flexible electronics, organic materials have been widely studied because of their high deformability and their capacity to perform certain sensitive functions. However, due to the different test principles, different structures and materials of the sensing units, and flexible substrates used, incompatibility often occurs, which causes a series of problems—such as complex structure, complicated preparation process, and poor reliability. In addition, carrier mobility is low, and the resistance change is lagging, so manufactured electronic devices have difficulty competing with the electronic devices built of more mature inorganic materials (such as Si). The brittleness and low ductility of inorganic materials seriously restrict the application in the field of flexible electronics. Compared to inorganic bulk materials, inorganic thin films may withstand some degree of bending deformation. The flexion rigidity of silicon nano-films with a thickness of 2 nm is 15 orders of magnitude less than that of a silicon plate with a thickness of 200 μm. From the perspective of mechanics, the silicon nano-films can be combined with almost any surface [10]. For example, the top silicon structure of an SOI wafer can be transferred to a pre-stretched PDMS substrate to realize a corrugated silicon nano-belt [11]. As a result, the researchers have proposed a variety of flexible mechanical and electrical sensors based on silicon nano-films. While achieving flexibility, silicon nano-films still have high carrier mobility and piezoresistive properties. For example, in view of the current problem that rigid silicon-based hydrophone cannot be flexible and conformal, silicon nano-films can be used as sensitive units, and flexible materials can be used as structures to make flexible hydrophones, which can realize the micro-nano-flexible heterogeneous integrated preparation of silicon nano-film sensitive units. It can fit with the surface of the unmanned underwater vehicle, realize the two-dimensional vector detection of underwater acoustic signals, perceive the underwater sound field environment information, and play an irreplaceable role in ocean exploration [12].

By converting traditional inorganic materials, replacing hard substrates with flexible substrates, the flexible materials are combined with inorganic semiconductor devices. This process is mainly performed by transferring the silicon nano-films on a flexible substrate. Transfer printing is an effective means of bonding flexible materials and inorganic semiconductors. In recent years, many scholars have used different sacrificial layers and different stamps to transfer silicon nano-films. Keon Jae Lee proposed in 2005 to incompletely etch the buried oxide layer of SOI wafers, with the residual buried oxide layer as fixed support, and use PDMS as the stamp to transfer print the silicon nano-films onto the PU-coated PET substrate. However, the films were broken during the process, and the structure was partially defective [13]. In 2006, Matthew A. Meitl et al. used the flat stamp to transfer the silicon nano-film structures. The method is simple and convenient, but the silicon nano-film structure transfer printing is relatively large, which is not suitable for very tiny devices [14]. Sang Il Park et al. used the PDMS stamp with relief structures on the surface to transfer the printing part of the dense array structure to other receiving substrates by embossing and optical alignment [15]. This selective transfer printing structure using the structured PDMS stamp increases the complexity of the subsequent process. Seok Kim et al. proposed, in 2010, that by removing the buried oxide layer of the SOI wafer, using photoresist as the fixed support for the silicon nano-films structure, the PDMS stamp with the micro-needle tip surface was placed on a precise three-dimensional displacement platform at a speed of 5 μm/s to collect the structure. Keep it under the specified load for 5 s, and then take it out at a speed of 1 mm/s. The transfer rate is close to 100%, and the minimum transfer size is 100 μm × 100 μm [16], which is not suitable for tiny silicon nano-films transfer. Yumi Yang proposed in 2011 to remove the buried oxide layer of the SOI wafer, align the photolithography a second time, and etch holes on the silicon nano-film structure, and transfer print to the tape by using PDMS in the form of a flat plate and a roller as a stamp, within a specific pressure range. The minimum structure of the transfer is 50 μm × 80 μm [17]. In 2015, Sheng Xu proposed to remove the buried oxide layer of the SOI wafer, align the photolithography a second time, fix and support the top silicon structure around each structure with PTFE, and transfer print utilizing contact formation of covalent bonds after ozone treatment of the ribbon-shaped silicon nano-films and Dragon Skin [18].

The above studies used the viscoelastic PDMS material as the stamp to transfer printing the silicon nano-films to other substrates. PDMS has been widely used in the field of MEMS. Combining PDMS and sensitive materials is a trend in developing MEMS. This paper proposes a method of transfer printing the top silicon structure of the SOI wafer to the PDMS substrate through the difference of adhesion between the PDMS stamp and the receiving substrate to the silicon nano-films. On this basis, a method is proposed to complete the transfer printing by forming an irreversible covalent bond between the silicon nano-films and PDMS after processing with oxygen plasma. Without the need for secondary alignment lithography and transfer print rate and pressure, flat stamp successfully transfers printed a 340 nm thick and 20 μm to 100 μm small rectangular pattern. The width of the pressure-sensitive unit of the existing rigid silicon-based hydrophone is 20 μm, which meets the size requirements of the pressure-sensitive unit of the hydrophone [19]. Furthermore, the progress has laid a foundation for combining silicon nano-films and PDMS materials in the MEMS field. In addition, most sensors using silicon as sensitive units—such as pressure sensors, accelerometers, microphones, inertial sensors, and other fields—can use this transfer printing method to produce the devices. This approach enables many sensors with flexible structures and silicon-based sensitive units replace rigid silicon-based sensors, overcoming the limitations of their application environments. This transfer printing method promotes the heterogeneous integration of flexible materials and semiconductors and plays a key role in developing the miniaturization of flexible sensors. In addition, this work can provide an important platform technology for which the simple-step of transfer printing of small structure of silicon nano-films for many tailored applications in flexible electronics.

## 2. Result and Discussion

### 2.1. Principle of Transfer Printing

The silicon nano-film is vital for the manufacturing of flexible hybrid electronics. Most of the materials used for preparing silicon nano-films consist of a hard silicon-based substrate, an intermediate sacrificial layer, and a top silicon nano-film. The silicon nano-films are released by removing the central sacrificial layer. The silicon nano-films must be separated from the hard silicon substrate before transfer printing. Subsequently, the different adhesion of the silicon-based substrate, the stamp, and the receiving substrate to the silicon nano-films are used to transfer print the structure. After the buried oxide layer of the SOI wafer is completely etched with hydrofluoric acid, there is no adhesion between the top silicon and the substrate silicon due to the supporting effect of the photoresist. At this time, using PDMS with a low base fluid to the curing agent ratio to stick down the silicon nano-films structure from the silicon substrate. When the curing temperature, time, environment, and other conditions are the same, the lower the proportion of the curing agent added, the stronger the surface adhesion of the cured PDMS to silicon nano-films [20]. Then, the PDMS with a high base fluid to the curing agent ratio adhered the silicon nano-films from the surface of the PDMS stamp.

### 2.2. Transfer Printing Process

**Figure 1:**
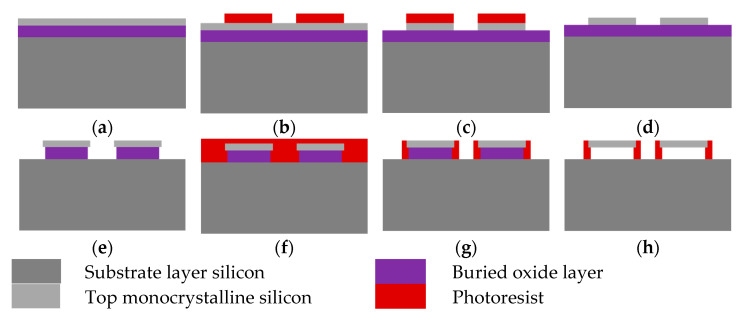
Sketch of the transferable silicon nano-films process: (**a**) SOI preparation; (**b**) first photolithography; (**c**) RIE etching; (**d**) removal of photoresist; (**e**) BOE corrosion; (**f**) coating photoresist; (**g**) second photolithography; (**h**) hydrofluoric acid etching.

The process of using the buried oxide layer of the SOI wafer as the sacrificial layer and the photoresist as the support to transfer print the silicon nano-films structure is shown in Figure 1. After patterning the top silicon of the SOI wafer by photolithography and RIE etching, the BOE solution is used to wet corrode a small part of the buried oxide layer at the edge of the silicon nano-films structure to form a roof-like structure and then spin-coating a layer of photoresist on the wafer. After photolithography and development without a photomask, the photoresist is retained at the roof. There are three reasons for applying photoresist for the second time here. First, without a fixed support, the silicon nano-films will be dislocated during the etching in the hydrofluoric acid and cleaning process. After the buried oxide layer is etched, the silicon nano-films will have strong adhesion to the substrate layer, which will have a certain impact on the transfer. In addition, other materials are used as support, such as the PTFE used earlier. Although the effect is the same as that of a photoresist, it needs to perform secondary alignment photolithography. The error will affect the role of PTFE in fixing and supporting the silicon nano-films. The last one, the radius of fluorine ion in hydrofluoric acid is very small, even smaller than that of oxygen ion, it is very permeable, and the dense oxides cannot prevent it from permeating. Therefore, hydrofluoric acid can pass through the photoresist to corrode the buried oxygen layer of the SOI wafer.

**Figure 2:**
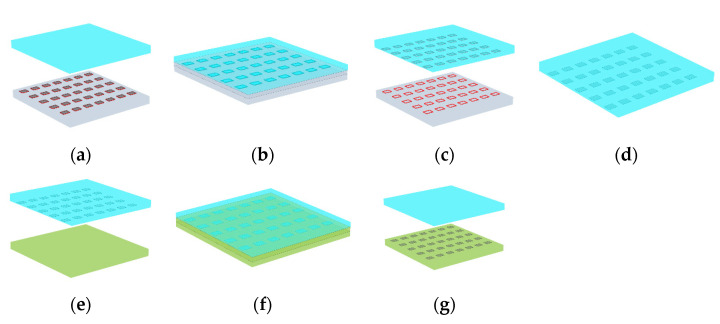
Schematic diagram of the silicon nano-films transfer printing steps: (**a**) PDMS A slowly approached the silicon nano-films; (**b**) PDMS A was in contact with the silicon nano-films; (**c**) the silicon nano-films were transfer printed from the SOI wafer to PDMS A; (**d**) remove the photoresist; (**e**) PDMS A slowly approached PDMS B; (**f**) PDMS A and the silicon nano-films contact PDMS B; (**g**) the silicon nano-films were printed to PDMS B.

At present, there are mainly three kinds of transfer methods commonly used—namely, transfer by means of a stamp with structures on the surface, transfer by a tangential force, and transfer by a shrinkable or expandable stamp. However, these three transfer methods require special operating instruments or stamps with special materials and structures or require transfer rate and stress [21], and many process details should be paid careful attention in actual operation. Therefore, this paper uses the difference of adhesion between the stamp and receiving substrate to the silicon nano-films to complete the transfer printing, which has no requirement for unique materials, structures, and instruments or need for stress and rate. Figure 2 shows the transfer process of the silicon nano-films structure. For the convenience of description, the PDMS stamp with the base fluid and the curing agent ratio of 5:1 is referred to as PDMS A, and the PDMS receiving substrate with the base fluid and the curing agent ratio of 10:1 is referred to as PDMS B. Figure 2a–d is the silicon nano-films transfer printing from the SOI wafer to PDMS A, and Figure 2e–g is the silicon nano-films transfer printing from PDMS A to PDMS B. The silicon nano-films are transferred from the SOI wafer to PDMS B by the PDMS A stamp. Based on this, silicon nano-films are combined with flexible PDMS to be applied in flexible electronics, achieving the requirements of flexibility, high sensitivity, high precision, low coupling, fast response, and high stability.

**Figure 3:**
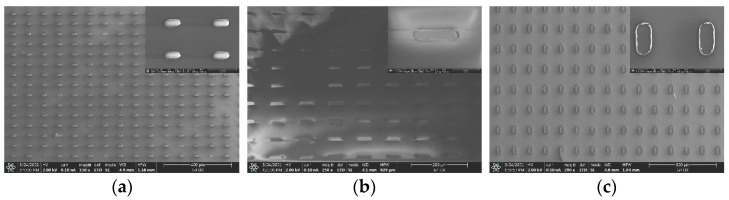
Transfer printing image of the silicon nano-films structure array: (**a**) silicon nano-films are on SOI after hydrofluoric acid etching; (**b**) silicon nano-films are transferred to PDMS B; (**c**) photoresist remaining on the SOI after the silicon nano-films are transferred.

The silicon nano-films transferred in this paper are a 40 μm × 20 μm rectangular array structure with a pitch of 50 μm. Figure 3a shows the silicon nano-films structure after the SOI wafer has completely etched the buried oxide layer. The prepared silicon nano-films structure has adhered to PDMS A, and the silicon nano-films are transferred from PDMS A to PDMS B. Figure 3b is the silicon nano-film structure transferred to PDMS B. There is a 99.3% transfer rate. Figure 3c shows the SOI wafer after the transfer. The residual photoresist on the wafer serves as a fixed support. Following tests and observations, most of the photoresist is transferred to PDMS A along with the silicon nano-films. Only a small part is left on the SOI wafer. The PDMS with the silicon nano-films were folded in half to observe that the silicon nano-films did not break. The reason is that although silicon nano-films have to bear a certain strain and very large stress when they are on the folded and bent PDMS. Nano silicon-films have a certain flexibility, they can adapt to a certain degree of bending and can fit to almost any surface, so they will not break. Therefore, the micro silicon nano-film structure can be suitable for large deformation structures.

**Figure 4:**
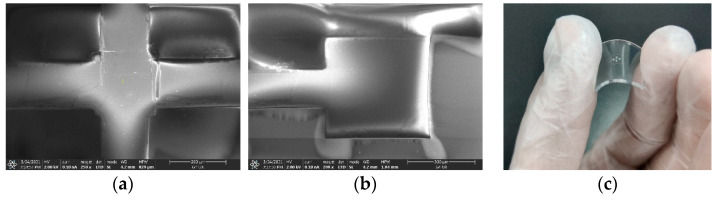
Comparison of silicon nano-films transfer to PDMS B: (**a**) silicon nano-films structure and PDMS B are transfer printed utilizing adhesion difference; (**b**) silicon nano-films structure and PDMS B are transfer printed by bonding; (**c**) bending of complex silicon nano-films structure on PDMS.

Figure 4 shows the comparison of silicon nano-films structure transferred to PDMS B by adhesion and bonding. Figure 4a shows the transfer print of the silicon nano-films structure and PDMS B through different viscosities, and Figure 4b shows the transfer print of the silicon nano-films structure and PDMS B through bonding to form an irreversible chemical bond. It can be observed that, after the complex and large-area silicon nano-films structure is transfer printed to the PDMS, a few cracks will occur. When bending a particular arc, the silicon nano-films do not separate from the PDMS, but if you keep bending, the silicon nano-films will break.

**Figure 5:**
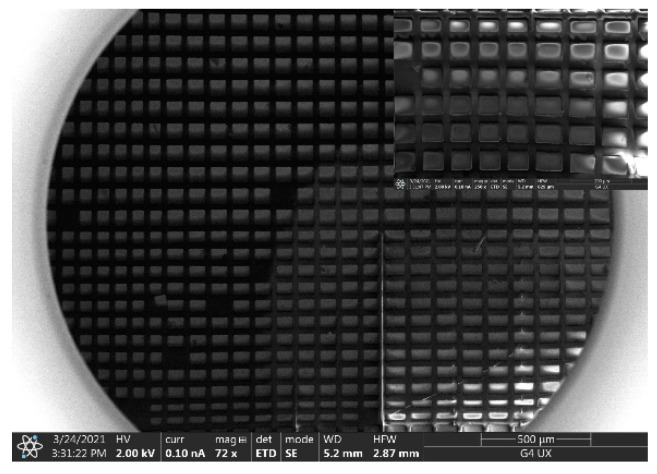
Silicon nano-films transfer printed to the PI substrate.

In addition, the silicon nano-films can also be transferred to other flexible substrates—such as PI, PET, etc.—through the stamp. The PDMS stamp with the silicon nano-films are transfer printed when close to the surface of the PI with a certain viscosity, which is not fully cured, then the PI is amidated. Figure 5 is the SEM image of the silicon nano-films transfer printed to PI, and the PI is entirely amidated. We can see that there are no obvious cracks in the silicon nano-films, and the transfer printing effect is good.

It is important to note that this method of transfer printing is easy to use. The structures of the silicon nano-films are supported and fixed by the photoresist. The PDMS base to the curing agent ratio that affects the adhesion to silicon and the silicon nano-films structure made of SOI wafer can be transfer printed to the receiving PDMS through the PDMS stamp, or the PDMS is transfer printed to the receiving PDMS by the way that the silicon nano-films and PDMS are processed to form an irreversible covalent bond. Furthermore, this transfer printing method does not need speed and pressure. It can transfer silicon nano-films structures of various sizes with a success rate of 99.3%, laying a good foundation for the development of flexible electronics in the future.

## 3. Materials and Methods

### 3.1. Fabrication of Silicon Nano-Film Structure

The silicon nano-films transferred in this paper is made of the SOI wafer (Silicon On Insulator). The thickness of top silicon is 340 nm, the thickness of the buried oxide layer is 3 μm, and the thickness of the substrate layer is 700 μm. First, a layer of HMDS (1,1,1,3,3,3-Hexamethyldisilazane) is applied to the SOI wafer as a bonding aid for the photoresist, and then a uniform spin-coating on the SOI wafer layer photoresist (AZ 6130 3 krpm/min 60 s + 4 krpm/min 5 s, about 2.5 μm thick), after exposure by photomask and photolithography machine (100 ${\mathrm{mJ}/\mathrm{cm}}^{2}$); use developer (AZ 400 K:H_2_O = 1:4, 25 s) to develop (40 µm × 20 µm rectangular structure with a pitch of 50 µm). Then RIE (reactive ion etching) is used to etch the exposed top silicon. Next, the BOE solution (hydrofluoric acid:H_2_O = 1:6) is used for wet corrosion of the SOI wafer, so that the top silicon structure and the central buried oxide layer form a roof-like structure. Subsequently, a photoresist layer (AZ 6130 5 krpm/min 60 s + 6 krpm/min 5 s, about 1.8 µm thick) is uniformly covered on the SOI wafer. The photoresist will also be filled in the roof-like structure. Exposed on the lithography machine (20 ${\mathrm{mJ}/\mathrm{cm}}^{2}$) without a photomask, use the developer (AZ 400 K:H_2_O = 1:3, 30 s) to develop, leave the photoresist in the roof-like structure, which can fix and support the top silicon structure and reduce the effect of the adhesion between the top silicon structure and the substrate silicon after the buried oxide layer is eradicated. Finally, the hydrofluoric acid (content: 48.0–50.0%) is used to completely engrave the remaining buried oxide layer of the SOI wafer. Finally, the SOI wafer is placed in deionized water to remove the residual hydrofluoric acid.

### 3.2. Fabrication of Stamp and Receiving Substrate

The stamp is made of PDMS (Polydimethylsiloxane, Dow Corning 184, Midland, MI, USA) base liquid and the curing agent with a mass ratio of 5:1. After mixing, keep stirring for 15 min. The mixture is then put into a vacuum drying oven for vacuum treatment until there are no more bubbles. A photoresist layer (AZ 6130 3 krpm/min 60 s, 3 krpm/min 5 s, about 2.5 µm thick) was uniformly spin-coated on a clean silicon wafer, and the films were hardened on a hot plate at 120 °C for 15 min. The vacuum-treated PDMS mixture is slowly poured onto the silicon wafer. The wafer is rotated at a low speed (0.5 krpm/min 5 s) so that the PDMS mixture completely covers the silicon wafer. The wafer is then placed in a vacuum drying oven for vacuum treatment to ensure that there are no bubbles in the PDMS. Finally, the wafer with PDMS on the surface is placed on a heating plate, and the temperature is slowly raised from room temperature to 75 °C, heated continuously for 3 h, and then slowly lowered to room temperature. The receiving substrate is made of PDMS base liquid and the curing agent with a mass ratio of 10:1, and stirred continuously for 15 min. The mixed solution is placed in a vacuum drying oven for vacuum treatment until the mixed liquid is totally free from bubbles. A clean wafer was put on the spin coater, and the PDMS mixture solution after vacuum treatment was slowly poured on the wafer. The wafer was spun at a low speed (0.5 krpm/min 5 s) to make the PDMS mixture completely cover the silicon wafer. The wafer was placed in a vacuum drying oven for vacuum processing to avoid bubbles in the PDMS. After that, the wafer was spun at high speed (0.5 krpm/min 5 s + 3 krpm/min 35 s, the thickness is about 20 µm). Finally, the wafer is placed on the heating plate, the temperature is slowly raised from room temperature to 75 °C, the heating is continued for 3 h, and finally, the temperature is gradually reduced to room temperature.

### 3.3. Transfer Printing

The process of transfer printing based on the difference in adhesion between the PDMS stamp and the receiving substrate is as follows. Slowly contact the fully cured PDMS A with the silicon nano-films structure on the SOI wafer. The adhesion of PDMS A surface is sufficient to adhere the silicon nano-films structure. Then, the PDMS A was peeled off the surface of the SOI wafer, and the PDMS A with the silicon nano-films structure adhered to it was immersed in an acetone solution for 5 s to remove the photoresist of the fixing and support structure and then immersed in deionized water to remove the residual acetone solution. The PDMS A with the silicon nano-films structure is immersed in the acetone solution for a very short time, and the PDMS areas where the silicon nano-films are located have no direct contact with the acetone solution, and the silicon nano-films can be attached to almost any surface. Therefore, the silicon nano-films are not affected by acetone solution, and they do not have any defects or cracks. Finally, PDMS A is slowly contacted with the cured PDMS B. Since the adhesion of PDMS B to silicon nano-films are greater than that of PDMS A, the silicon nano-films structure can be transferred from PDMS A to PDMS B. When transferring by bonding method, it is necessary to put PDMS A with silicon nano-films structure and PDMS B receiving silicon nano-films structure into a plasma stripper for 10 s (O_2_ 300 sccm, 400 W), quickly contact and bond the silicon structure on PDMS A with PDMS B, and the PDMS A stamp was peeled off. Then put PDMS B on a heating plate at 100 °C for 30 min so that the silicon structure and PDMS bond more firmly.

The transfer printing of the silicon nano-films to the PI receiving substrate by the PDMS stamp is as follows. Transfer print the silicon nano-films to PDMS stamp first, and then put it respectively in acetone solution and deionized water for 5 s. Spin-coat PI reagent on the silicon wafer, bake it on a heating plate at 115 °C for 60 s and then remove the wafer for transfer. After the transfer printing, the wafer is placed on the heating plate for 120 s and then amidated (80 °C 0.5 h, 140 °C 1 h, 300 °C 1.5 h).

## 4. Conclusions

This paper proposes a method that uses the buried oxide layer of the SOI wafer as the sacrificial layer, the photoresist fixes and supports the top silicon nano-films structure. The PDMS base to the curing agent ratio affects the adhesion to silicon and enables the transfer. The method of transferring silicon nano-films structure through the PDMS stamp to the PDMS or PI receiving substrate has no requirement for secondary alignment photolithography and can transfer 20 μm-wide structures. Experiments prove that this transfer printing method is feasible. PDMS and PI are popular materials in flexible electronics, and silicon nano-films can be bent to a certain extent. Batch transfer of micro-structured silicon nano-films will help to reduce the structural size of flexible microelectronic products and improve the degree of integration. In the future, PDMS and PI films can be patterned as flexible structures, combined with silicon nano-films as the sensitive units, and applied to flexible electronics.
